# Prosthetic Joint Infection from Carbapenemase-Resistant *Klebsiella pneumoniae* Successfully Treated with Ceftazidime-Avibactam

**DOI:** 10.1155/2018/1854805

**Published:** 2018-08-14

**Authors:** A. Schimmenti, E. Brunetti, E. Seminari, B. Mariani, P. Cambieri, P. Orsolini

**Affiliations:** ^1^Dipartimento di Scienze Clinico-Chirurgiche, Diagnostiche e Pediatriche, Unità di Malattie Infettive e Tropicali ed Epatologia, Università di Pavia, Pavia, Italy; ^2^Unità di Malattie Infettive e Tropicali, Fondazione IRCCS Policlinico San Matteo, Pavia, Italy; ^3^Microbiologia e Virologia, Fondazione IRCCS Policlinico San Matteo, Pavia, Italy; ^4^Dipartimento di Medicina Interna e Terapia Medica, Università di Pavia, Pavia, Italy

## Abstract

Antimicrobial resistance in Gram-negative bacteria, particularly Enterobacteriaceae, has become a leading cause of morbidity and mortality and a serious public health concern. Gram-negative bacteria carrying extended-spectrum beta-lactamase (ESBL) enzymes now represent a significant proportion of all bacteria isolated from different countries worldwide. Furthermore, the increasing number of isolates carrying carbapenemases in recent years includes multidrug-resistant (MDR), extensively drug-resistant (XDR), and pandrug-resistant (PDR) bacteria. Here, we describe what, to our knowledge, is the first case of a patient with a prosthetic joint infection from carbapenemase-resistant *Klebsiella pneumoniae* (CRKP) successfully treated with ceftazidime-avibactam in Italy.

## 1. Introduction

Antimicrobial resistance in Gram-negative bacteria, particularly Enterobacteriaceae, has become a leading cause of morbidity and mortality and a serious public health concern [1]. Gram-negative bacteria carrying extended-spectrum beta-lactamase (ESBL) enzymes now represent a significant proportion of all bacteria isolated from different countries worldwide [1]. The emergence and spread of carbapenemase-producing pathogens (including carbapenem-resistant *Acinetobacter baumannii*, *Pseudomonas aeruginosa*, and Enterobacteriaceae) is thus of particular concern and has highlighted the urgent need for new antimicrobial agents [2].

In Europe, the antibiotic resistance of *K. pneumoniae* is a problem, especially in a group of southern and eastern countries. In Italy, the percentage of invasive isolates of *K. pneumoniae* with resistance to aminoglycosides was between 25 and 50% and that of isolates with resistance to third-generation cephalosporins was over 50% in a 2017 document from the ECDC [3].

Aminoglycosides maintain a potent bactericidal activity against some, not all, carbapenem-resistant Enterobacteriaceae (CRE) [4]. Data about this are relatively scarce in the literature. In a paper by a group from China [5], the resistance rates of CRE to aminoglycosides varied from 14.7% to amikacin to 81.7% to gentamicin and, based on the reference method, are in line with previous studies [6, 7]. In another study, nonsusceptibility rates of gentamicin, tobramycin, and amikacin were 40, 98, and 16%, respectively [4].

Here, we describe a patient with a prosthetic joint infection from carbapenemase-resistant *Klebsiella pneumoniae* (CRKP) successfully treated with ceftazidime-avibactam in a hospital in Northern Italy.

## 2. Case Presentation

A 26-year-old Italian Caucasian male had a trauma from a fall on July 2014, with multiple fractures including left hemipelvis with luxation of coxofemoral joint (managed with reduction and osteosynthesis of the posterior acetabular wall), distal third of the right femur (treated with an osteosynthesis with plate and screws), and distal diaphysis of the right fibula (osteosynthesis plate and screws) along with facial skull trauma and chest trauma. All surgeries were executed in late 2014, and only perioperative antibiotic prophylaxis had been administered.

He also had a history of fracture of the left femur at 11 years of age treated with osteosynthesis with a rod that was subsequently removed, Von Willebrand disease, and depressive disorder.

On July 11, 2016, he was admitted to orthopedic surgery for redness and swelling of the right knee joint with a fistula on the right distal limb.

**Figure 1:**
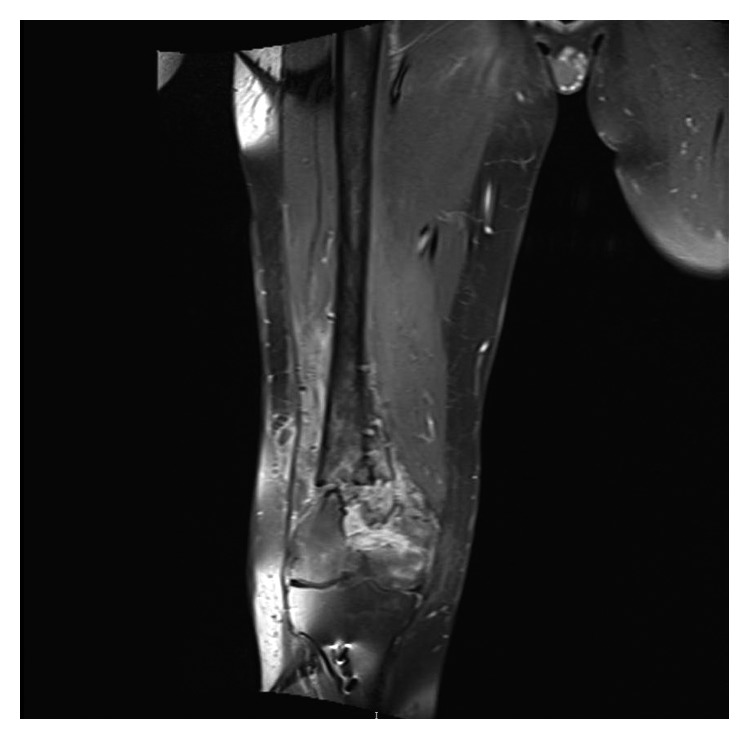
MRI of the the right knee and femur showing that the bone tissue is heavily disrupted with several infectious lytic foci in the diaphyseal and metaphyseal portion, swelling of the cancellous bone, and periosteal reaction. A cutaneous fistula draining from a fluid collection in the vastus lateralis muscle can be seen.

Magnetic resonance imaging (MRI) of the right knee and femur showed osteomyelitis of the distal femur (Figure 1).

The patient underwent surgery with removal of implants, a fistulectomy of the right femur was performed, biopsies were collected, sonication of the plate was performed, and a knee brace was placed.

The same CRKP strains were isolated both on cultures of biopsies and on prosthetic material after sonication. Bacterial identification and antimicrobial susceptibility testing were performed using the Phoenix Automated Microbiology System (Becton Dickinson Diagnostic Systems, Sparks, MD, USA). Confirmatory MIC testing for imipenem and meropenem was carried out by gradient test for MIC determination (Etest Liofilchem, Roseto degli Abruzzi, Italy) and interpreted in accordance with the European Committee on Antimicrobial Susceptibility Testing (EUCAST) breakpoints [8].

The *Klebsiella pneumoniae* isolate was further evaluated for the presence of carbapenemase using a phenotypic assay (Rosco, Stamford, CT, USA) containing discs of meropenem (10 *μ*g), meropenem + phenyl boronic acid (PBA), meropenem + dipicolinic acid (DPA), and meropenem + cloxacillin (CL). The organism was confirmed as a class A (KPC) carbapenemase enzyme producer. In detail, KPC enzymes are inhibited by phenylboronic acid.

A real-time PCR, detecting several genes involved in carbapenem resistance (Xpert Carba-R; Cepheid, Sunnyvale, CA, USA), was performed to confirm the results of the phenotypic test. More specifically, this method allows for the detection and differentiation of the most frequent carbapenemases gene families (*bla*_*KPC*_, *bla*_*VIM*_, *bla*_*OXA-48*_, *bla*_*IMP-1*_, and *bla*_*NDM*_) in Gram-negative bacteria. The real-time PCR resulted positive for KPC and negative for VIM, OXA-48, IMP-1, and NDM.

**Figure 2:**
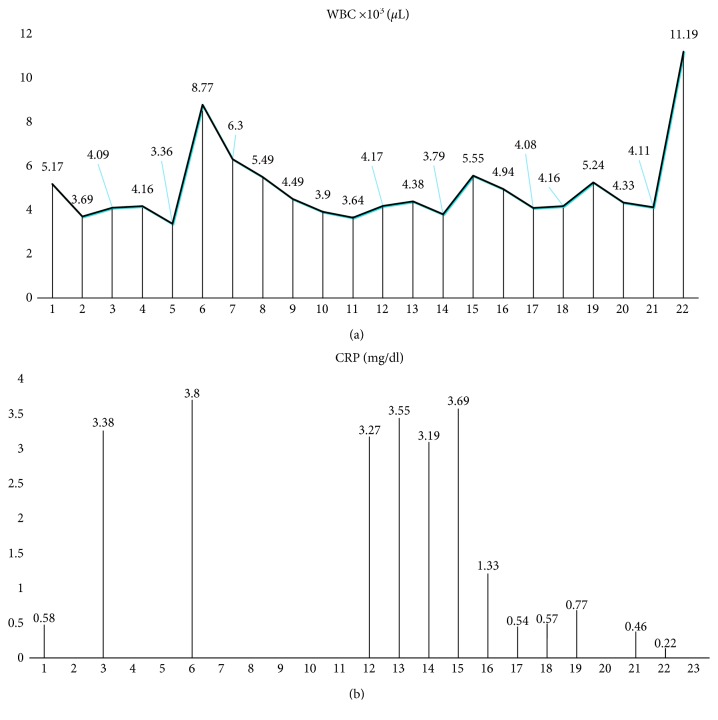
Kinetics of WBC and acute phase reactant during the clinical course of infection.

**Figure 3:**
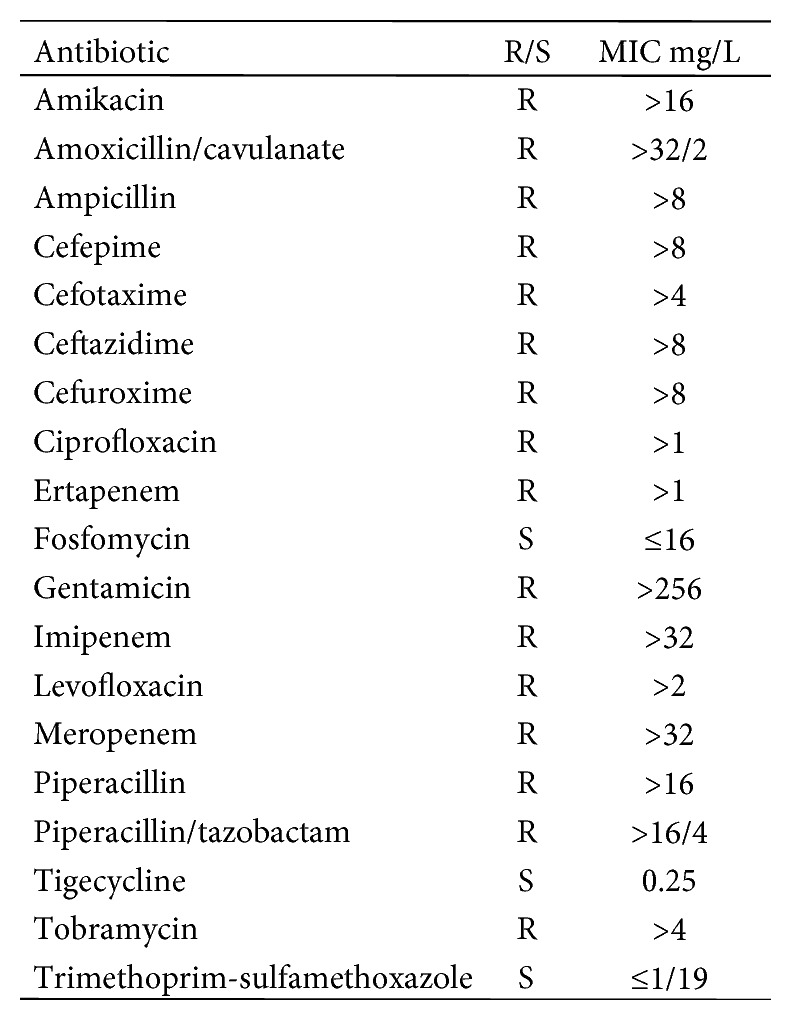
Antibiotic susceptibility according to the European Committee on Antimicrobial Susceptibility Testing (EUCAST) clinical breakpoints of clinical *Klebsiella pneumonia* isolate. MIC: minimum inhibitory concentration; R: resistant; S: susceptible.

On July 20th, the patient was started on colistin-fosfomycin and trimethoprim-sulfamethoxazole and then transferred to the Infectious Disease (ID) ward. We switched antibiotic therapy to colistin (loading dose of 9 million IU, and then 4.5 million IU BID), fosfomycin (4 g every eight hours), and tigecycline (loading dose of 100 mg, and then 50 mg BID). After the switch, he reported frequent nausea, while renal function remained normal and acute phase reactants remained elevated.  Figure 2 shows the kinetics of white blood cells and acute phase reactants, and Figure 3 the antibiotics susceptibility test.

On August 4th, the patient was transferred to Orthopedic Surgery for resection of the distal femur along with minimal resection of the proximal fibula with positioning of a cemented Stage one® (Zimmer Biomet, Warsaw, IN, USA) spacer with an intramedullary rod in the femur. Samples of both bones were cultured, and tissue collected during surgery was negative. On August 5, the patient was transferred back to ID ward, and because of the onset of fever, poor tolerance of antibiotic therapy, and increase of acute phase reactants, we requested susceptibility testing for ceftazidime/avibactam (C/A) to our Bacteriology Laboratory. Sensitivity to C/A was confirmed using the specific disc (BD) provided by AstraZeneca, Molndal, Sweden. Resistance to carbapenems was further confirmed with the Xpert® Carba-R molecular diagnostic system (Cepheid, Sunnyvale, CA, USA). On August 19 after approval from our ethics committee for off-label use, we started treatment with ceftazidime/avibactam at a dose of 2.5 g TID for 2 weeks. In the subsequent days, the patient's clinical condition and laboratory tests improved with healing of the wound except for a fistula in the middle of the wound (fistula and rectal swabs were negative for CRKP). On September 2, a technetium-99 bone scan was performed showing distal uptake in the site of surgical intervention (which was deemed normal given that less than 12 months had elapsed from surgery). On September 16, the patient was transferred to the Orthopedic Surgery ward for a surgical curettage of the fistula. After being transferred back to the ID ward, the patient remained afebrile and daily care of the wound showed no discharge and no fibrosis. Cultures of samples taken during the curettage were negative. On October 14, the cemented spacer was removed in the Orthopedic Surgery ward and definitive knee prosthesis was positioned.

**Figure 4:**
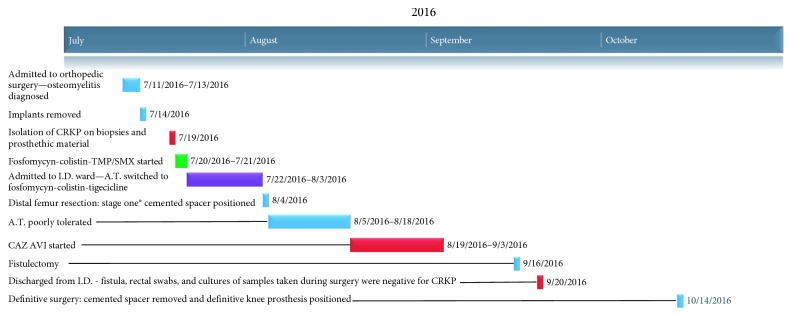
Timeline of antibiotic and surgical treatments.

The patient was discharged from the ID ward on September 20, 2016.  Figure 4 shows a timeline reviewing the events presented in this case report. During the latest orthopedic follow-up visit on February 23, 2017, the patient had no signs and symptoms of infection, was walking with the help of crutches, and continued being treated with physical therapy.

## 3. Discussion

The increased number of isolates carrying carbapenemases seen in recent years constitutes a challenge, as it spans multidrug-resistant (MDR), extensively drug-resistant (XDR), and pandrug-resistant (PDR) bacteria [8, 9].

Management of carbapenem-resistant Enterobacteriaceae (CRE) infections is limited by the paucity of effective treatment options. Prior to 2015, frontline regimens included combinations of agents with high toxicity rates (aminoglycosides and colistin), suboptimal pharmacokinetics (aminoglycosides, colistin, and tigecycline), and/or known microbiological resistance (carbapenems). In February 2015, the U.S. Food and Drug Administration (FDA) approved ceftazidime-avibactam (C-A), a novel beta-lactam/lactamase inhibitor with *in vitro* activity against CRE expressing *Klebsiella pneumoniae* carbapenemases (KPCs) but not Ambler class B or some class D lactamases [10]. Ceftazidime-avibactam has the same spectrum of activity as ceftazidime (e.g., against Enterobacteriaceae and *P. aeruginosa*), but the addition of avibactam expands its activity against many resistant strains carrying certain beta-lactamases [11].

Here, we report a case of a prosthetic joint infection due to KPC in a young male. In a Medline literature search using the keywords “ceftazidime-avibactam,” and “*Klebsiella pneumoniae*” and“prosthetic joint infection,” we found no items. When we used only “Klebsiella pneumoniae” and “prosthetic joint infection” we could find 9 references: of which, 4 [12–15] reported a total of 7 patients with a PJI from KP, but none from a CRKP. Of these, only a case series by de Sanctis et al. [16] reported three patients with PJI from CRKP. In their case series (1) the CRKP possessed blaKPC and were difficult to eradicate (persistent) in prosthetic joint infection; (2) multiple surgeries and antibiotic courses were undertaken and patients required a prolonged length of stay; (3) resistance to colistin and amikacin emerged on therapy; (4) the same strain of CRKP may be responsible for relapse of infection; and (5) significant morbidity and mortality resulted. Specifically, two patients died and one survived with major disability. Although our patient had significant comorbidities, his young age may have positively influenced his outcome. To our knowledge, this is the first case described in our country of a CRKP prosthetic joint infection successfully treated with ceftazidime/avibactam.

## References

[1] Alatoom A., Elsayed H., Lawlor K. (2017). Comparison of antimicrobial activity between ceftolozane-tazobactam and ceftazidime-avibactam against multidrug-resistant isolates of *Escherichia coli*, *Klebsiella pneumoniae*, and *Pseudomonas aeruginosa*. *International Journal of Infectious Diseases*.

[2] Shirley M. (2018). Ceftazidime-avibactam: a review in the treatment of serious gram-negative bacterial infections. *Drugs*.

[3] European Centre for Disease Prevention and Control (2017). *Surveillance of Antimicrobial Resistance in Europe 2016, Annual Report of the European Antimicrobial Resistance Surveillance Network (EARS-Net)*.

[4] Almaghrabi R., Clancy C. J., Doi Y. (2014). Carbapenem-resistant *Klebsiella pneumoniae* strains exhibit diversity in aminoglycoside-modifying enzymes, which exert differing effects on plazomicin and other agents. *Antimicrobial Agents and Chemotherapy*.

[5] Zhao Z., Lan F., Liu M. (2017). Evaluation of automated systems for aminoglycosides and fluoroquinolones susceptibility testing for Carbapenem-resistant Enterobacteriaceae. *Antimicrobial Resistance and Infection Control*.

[6] Hu L., Zhong Q., Shang Y. (2014). The prevalence of Carbapenemase genes and plasmid-mediated quinolone resistance determinants in carbapenem-resistant Enterobacteriaceae from five teaching hospitals in central China. *Epidemiology and Infection*.

[7] Xu A., Zheng B., Xu Y. C., Huang Z. G., Zhong N. S., Zhuo C. (2016). National epidemiology of carbapenem-resistant and extensively drug-resistant Gram-negative bacteria isolated from blood samples in China in 2013. *Clinical Microbiology and Infection*.

[8] EUCAST (2018). *Breakpoint Tables for Interpretation of MICs and Zone Diameters Version 80*.

[9] Magiorakos A. P., Srinivasan A., Carey R. B. (2012). Multidrug-resistant, extensively drug-resistant and pandrug-resistant bacteria: an international expert proposal for interim standard definitions for acquired resistance. *Clinical Microbiology and Infection*.

[10] Shields R. K., Nguyen M. H., Chen L. (2017). Ceftazidime-avibactam is superior to other treatment regimens against carbapenem-resistant *Klebsiella pneumoniae* Bacteremia. *Antimicrobial Agents and Chemotherapy*.

[11] Goodlet K. J., Nicolau D. P., Nailor M. D. (2016). Ceftolozane/tazobactam and ceftazidime/avibactam for the treatment of complicated intra-abdominal infections. *Therapeutics and Clinical Risk Management*.

[12] Pepke W., Lehner B., Bekeredjian-Ding I., Egermann M. (2013). Haematogenous infection of a total knee arthroplasty with *Klebsiella pneumoniae*. *Case Reports*.

[13] Tseng S. W., Chi C. Y., Chou C. H. (2012). Eight years experience in treatment of prosthetic joint infections at a teaching hospital in Central Taiwan. *Journal of Microbiology, Immunology and Infection*.

[14] Martínez-Pastor J. C., Vilchez F., Pitart C., Sierra J. M., Soriano A. (2010). Antibiotic resistance in orthopaedic surgery: acute knee prosthetic joint infections due to extended-spectrum beta-lactamase (ESBL)-producing Enterobacteriaceae. *European Journal of Clinical Microbiology and Infectious Diseases*.

[15] Chodos M. D., Johnson C. A. (2009). Hematogenous infection of a total knee arthroplasty with *Klebsiella pneumoniae* in association with occult adenocarcinoma of the cecum. *Journal of Arthroplasty*.

[16] de Sanctis J., Teixeira L., van Duin D. (2014). Complex prosthetic joint infections due to carbapenemase-producing *Klebsiella pneumoniae*: a unique challenge in the era of untreatable infections. *International Journal of Infectious Diseases*.

